# Dynamical density delay maps: simple, new method for visualising the behaviour of complex systems

**DOI:** 10.1186/1472-6947-14-6

**Published:** 2014-01-18

**Authors:** Anton Burykin, Madalena D Costa, Luca Citi, Ary L Goldberger

**Affiliations:** 1 Wyss Institute for Biologically Inspired Engineering at Harvard University, Boston, MA 02115, USA; 2 Margret and H.A. Rey Institute of Nonlinear Dynamics in Physiology and Medicine, Division of Interdisciplinary Medicine and Biotechnology, Beth Israel Deaconess Medical Center, Harvard Medical School, Boston, MA 02215, USA; 3 School of Computer Science and Electronic Engineering, University of Essex, Wivenhoe Park, Colchester CO4-3SQ, UK

**Keywords:** Atrial fibrillation, Delay map, Heart rate variability, Nonlinear dynamics, Poincaré plot, Sleep, Time series, Visualisation

## Abstract

**Background:**

Physiologic signals, such as cardiac interbeat intervals, exhibit complex fluctuations. However, capturing important dynamical properties, including nonstationarities may not be feasible from conventional time series graphical representations.

**Methods:**

We introduce a simple-to-implement visualisation method, termed *dynamical density delay mapping (“D3-Map”* technique*)* that provides an animated representation of a system’s dynamics. The method is based on a generalization of conventional two-dimensional (2D) Poincaré plots, which are scatter plots where each data point, *x(n),* in a time series is plotted against the adjacent one, *x(n + 1)*. First, we divide the original time series, *x(n)* (*n* = 1,…, *N*), into a sequence of segments (windows). Next, for each segment, a three-dimensional (3D) Poincaré surface plot of *x(n), x(n* + *1*)*, h*[*x(n),x(n* + *1)*] is generated, in which the third dimension, *h,* represents the relative frequency of occurrence of each (*x(n),x(n* + *1)*) point. This 3D Poincaré surface is then chromatised by mapping the relative frequency *h* values onto a colour scheme. We also generate a colourised 2D contour plot from each time series segment using the same colourmap scheme as for the 3D Poincaré surface. Finally, the original time series graph, the colourised 3D Poincaré surface plot, and its projection as a colourised 2D contour map for each segment, are animated to create the full “D3-Map.”

**Results:**

We first exemplify the D3-Map method using the cardiac interbeat interval time series from a healthy subject during sleeping hours. The animations uncover complex dynamical changes, such as transitions between states, and the relative amount of time the system spends in each state. We also illustrate the utility of the method in detecting hidden temporal patterns in the heart rate dynamics of a patient with atrial fibrillation. The videos, as well as the source code, are made publicly available.

**Conclusions:**

Animations based on density delay maps provide a new way of visualising dynamical properties of complex systems not apparent in time series graphs or standard Poincaré plot representations. Trainees in a variety of fields may find the animations useful as illustrations of fundamental but challenging concepts, such as nonstationarity and multistability. For investigators, the method may facilitate data exploration.

## Background

Physiologic and physical systems often generate highly complex output signals. An inherent feature of these signals is nonstationarity, defined as changes in their statistical properties (mean, variance, and higher moments) over time [1]. Furthermore, nonstationarities are important because they may contain information of both basic and translational interest. However, conventional representations, such as time series graphs, may not fully capture these time-varying properties. This challenge motivates the development of alternative ways to visualise the dynamics of complex time series.

We present a simple, new visualisation approach, based on the concept of delay maps, which highlights nonstationarities and related features. A classical delay map is the Poincaré plot^a^[1], widely used in biomedicine by investigators probing heart rate variability [2]. Our dynamical density delay map method, termed *D3-Map*, extends this concept to generate animated, colourised two and three-dimensional representations.

This paper is intended to serve both as a theoretical introduction and a practical tutorial so that readers can recreate all the figures and animations using our data or their own. Therefore, all videos, data and source code are made publicly available (http://reylab.bidmc.harvard.edu/.D3Map/).

Although Poincaré plots are among the simplest representation of a system’s phase space [1], they may provide important information about the dynamics. For illustration purposes here we use heartbeat time series, i.e., a sequence of consecutive ventricular interbeat (QRS) intervals (termed RR) intervals obtained from an electrocardiographic (ECG) recording [2]. However, our method is applicable to any time series of sufficient length.

**Figure 1:**
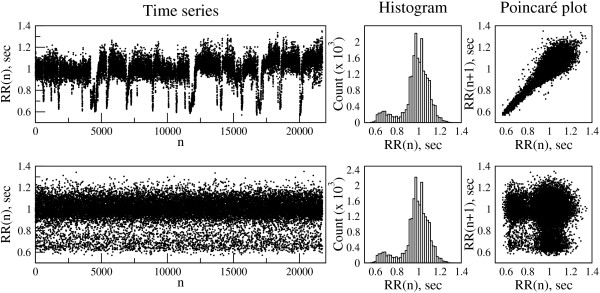
**Time series graph (left), histogram (center) and Poincaré plot (right) of the interbeat interval [RR(n)] time series derived from an electrocardiographic recording of a healthy subject during sleep (top) and from a randomized/shuffled sequence of these intervals (bottom).** Note that the histogram of the shuffled time series is identical to that of the original one, while the Poincaré plot is substantially altered (see text).

A Poincaré plot (map) of a time series is the scatter plot of *x(n)* versus *x(n + 1)* or equivalently, *x(n-1)* versus *x(n)*, hence the general term *delay map*^b^[1-7]. In Figure 1 (top), we show the RR interval time series from a healthy subject during sleeping hours, along with its histogram and the standard (“black and white”) Poincaré plot. As described further below, this dataset was selected because it exhibits a common type of nonstationarity characterized by relatively abrupt changes of “state”. The “comet-like” shape of the Poincaré plot of the RR interval time series (Figure 1, top) indicates a statistical relationship between consecutive data points where a given RR interval is likely followed or preceded by one of similar duration. In fact, the spread of the data points in the direction perpendicular to the diagonal of the Poincaré plot is a measure of the time series’ local variance (“short term variability”).

To highlight the added information that Poincaré plots provide, we shuffled (randomised) the order of the RR intervals. The histogram for this shuffled time series is unchanged from that of the original signal. However, the Poincaré plot is dramatically altered by the breakdown of correlations caused by the randomisation procedure (Figure 1, bottom).

**Figure 2:**
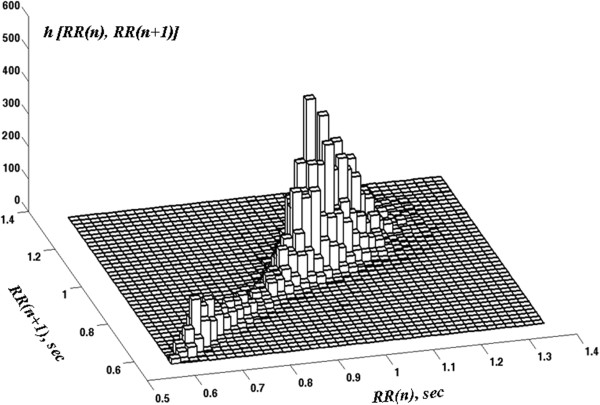
**2D histogram of RR(n), RR(n + 1) for the time series presented in the top panel of Figure **1.

This example illustrates how the standard Poincaré plot resolves one limitation of the histogram, namely that the latter does not provide information about *correlations* among the data points. However, standard (“black and white”) Poincaré plots have a salient limitation of their own; namely, they do not capture information about the density of the data points. This limitation can be overcome by modifying the standard Poincaré plot to include the relative frequency of pairs of consecutive data points by constructing a 2D histogram^c^ of RR(n), RR(n + 1) (Figure 2).

However, even this so-called “3D Poincaré plot” omits information about the *time evolution* of a system’s dynamics. For example, consider a time series from a dynamical system with two states, A and B. The 3D Poincaré plot constructed from the entire time series will be the same whether the system spends the first half of the time in state A and the second half in state B, or whether the system changes states on a much shorter time scale. Thus, the 3D Poincaré plot for the entire time series does not capture the *time sequence* of state changes. This limitation is particularly important when probing the dynamics of physiologic systems, which are typically non-stationary. Here, we introduce a visualization technique, termed D3-Map, based on a moving window analysis of the data.

## Methods

The D3-Map approach consists of five simple steps:

I. Divide the initial time series into a sequence of either overlapping or non-overlapping segments. Here, we analyse overlapping segments selected using a 500 sec moving window, shifted 10 sec at a time.

II. For each data segment, construct a normalised 2D histogram of RR(n) *vs* RR(n + 1) and its contour map. Let *m* be the number of counts in the highest bin of the histogram. The normalisation procedure consists of dividing the number of counts in each bin of the histogram by *m.* Here, we also opted for smoothing the histogram, using the dscatter^d^ Matlab function with the default parameter, which implements the method described in [8].

III. Colourise both the normalized 2D histogram and its contour map. In this implementation, we selected the *jet* colourmap,^e^ where blue represents the lowest and dark red-brown the highest density values, respectively. The colourised, normalised and smoothed 2D histogram of RR(n), RR(n + 1) is called the 3D *surface Poincaré plot*.

IV. In a single figure, present the 3D surface Poincaré plot, its colourised contour map^f^ and the graph of the original data segment used to generate them.

V. Sequentially present the figures with the analysis of each data segment as an animation [5]. Here, we used free video editing software VirtualDub (http://www.virtualdub.org) to create AVI movies and animated GIF images.^g^

The cardiac interbeat interval time series used in the examples were from open-access, deidentified data freely available at the NIH-sponsored PhysioNet website (http://www.physionet.org) [9]. The links to the specific PhysioNet data sets used are indicated in parenthesis in the text below.

## Results and discussion

The D3-Map visualisation method was first applied to the cardiac interbeat interval time series from the healthy subject whose data (Additional file 1) are shown in Figures 1 and 2. All figures presented here for this subject were generated from the same data set (nsr001 at http://physionet.org/physiobank/database/nsr2db/), which we chose because it exhibits a type of nonstationarity characterised by relatively abrupt state transitions, which are commonly observed in the output of “free-running” physiologic signals.

**Figure 3:**
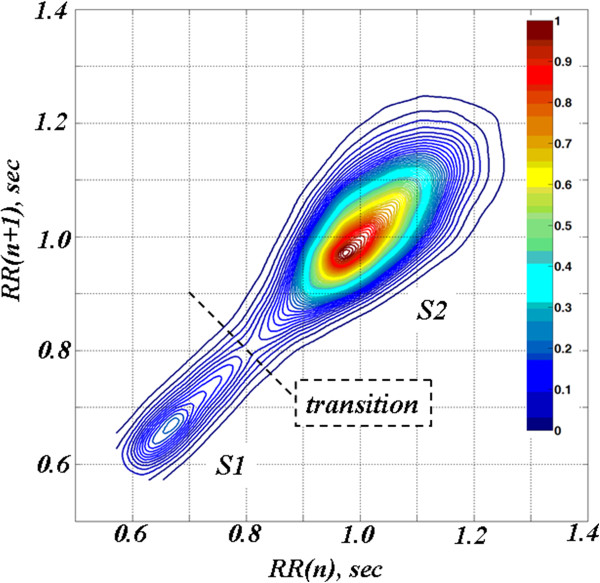
**Colourised contour map for the time series presented in the top panel of Figure **1. The relative density of each pair of points (RR(n), RR(n + 1)) is represented using the *jet* colourmap. Two states, S1 and S2 (local density maxima), separated by a dashed line drawn through the region of minimum density, are discernable.

In this specific case, (Figure 3, colourised 2D contour plot), there are two states (termed S1 and S2), centered around RR intervals of 0.65 sec and 1 sec (corresponding to periods of faster and slower heart rates of about 92 beats/min and 60 beats/min, respectively). These two states likely correspond to different sleep stages [10]: the faster rates to periods of rapid eye movement (REM) sleep (or transient awakenings) and the slower rates to deeper sleep. A movie of the 2D contour plots (Animation 1, Sleep2D.avi) providing information about the nature (rapid or gradual) of the transitions between the two states is included in the supplemental material. From a physiologic point of view, the “flipping” between these two states represents a highly nonstationary behavior, reminiscent of *bistability*[11].

**Figure 4:**
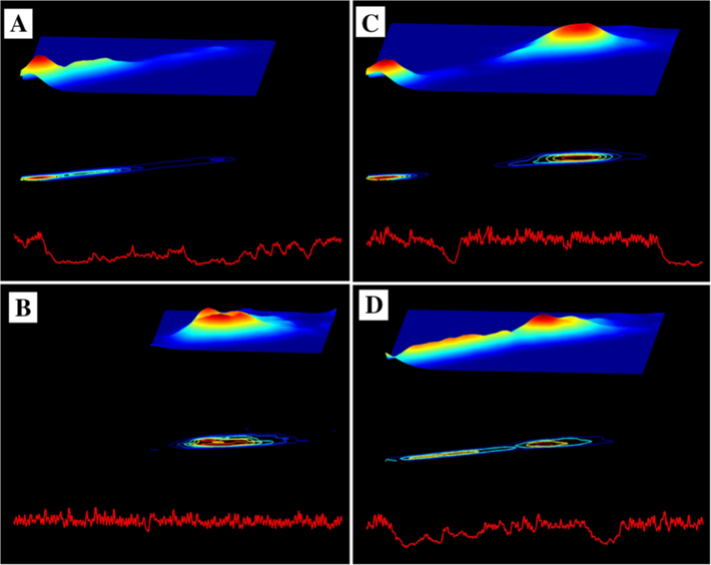
**Selected snapshots from the D3-Map animation derived from the analysis of the interbeat interval time series shown in the top panel of Figure **1. Each snapshot comprises: a 500 sec interbeat interval time series segment (bottom), its colourised 2D contour map (middle) and colourised 3D surface Poincaré map (top). (See text for details).

Figure 4 shows “snapshots” of the D3-Map animation included in the online supplementary material (Animation 2, Sleep3D.avi) for four different data segments. These snapshots demonstrate system transitions between different states over time. The D3-Map animations show that the states, themselves, are not static. Instead they appear to consist of multiple sub-states, manifesting as local undulations in the 3D surface, which continuously emerge and disappear. This finding is not apparent from visual inspection of the raw interbeat interval time series.

**Figure 5:**
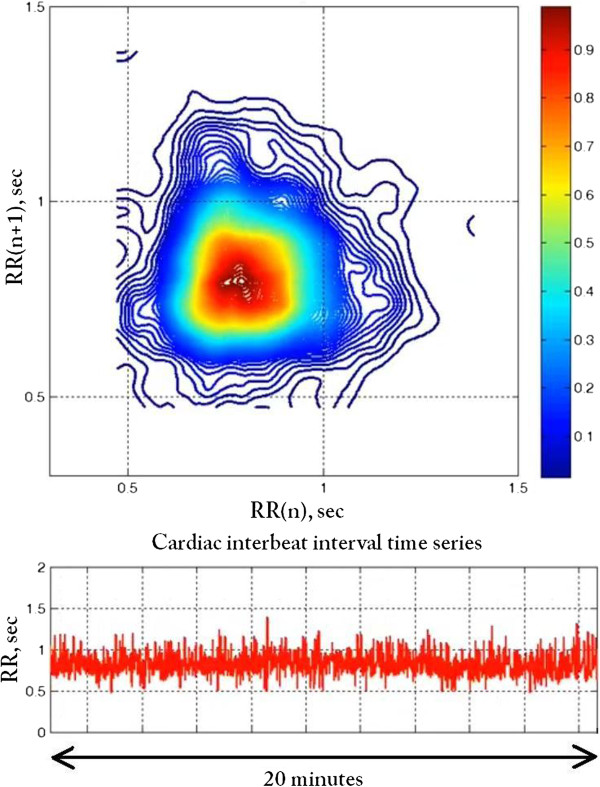
**Colourised contour map (top) of the RR interval time series (bottom) from a patient with typical pattern of atrial fibrillation.** The circular shape of the contour map is consistent with uncorrelated dynamics.

**Figure 6:**
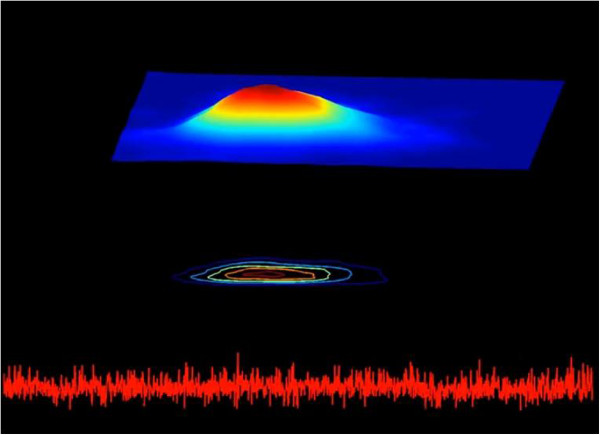
**Snapshot from the D3-Map animation derived from the analysis of a 1200 sec RR interval time series segment of the patient with a typical atrial fibrillation.** Top: colourised 3D surface Poincaré map; middle: colourised 2D contour map; bottom: interbeat interval time series.

To further explore the utility of this visualisation method, we also show the analysis of cardiac interbeat interval time series from two patients with chronic atrial fibrillation, a common cardiac arrhythmia that may be associated with stroke or heart failure. The RR dynamics during atrial fibrillation are presumed to be random. In fact, clinicians use the expression “irregularly irregular” to describe the unpredictable timings of the heartbeat during this arrhythmia. This assumption is supported by the circular shape of the contour map in Figure 5 from the first of these subjects (dataset a1nn from http://www.physionet.org/challenge/chaos/), which is typical of time series of uncorrelated values. The D3-Map movie (snapshot shown in Figure 6) shows that in this case, the dynamics is relatively stationary.

**Figure 7:**
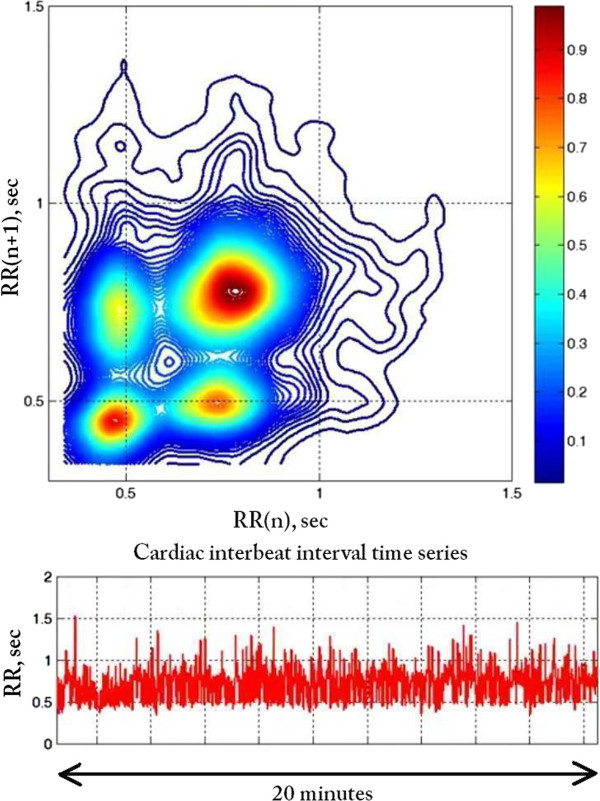
Colourised contour map (top) of the RR interval time series (bottom) from a patient with an atypical pattern of atrial fibrillation.

**Figure 8:**
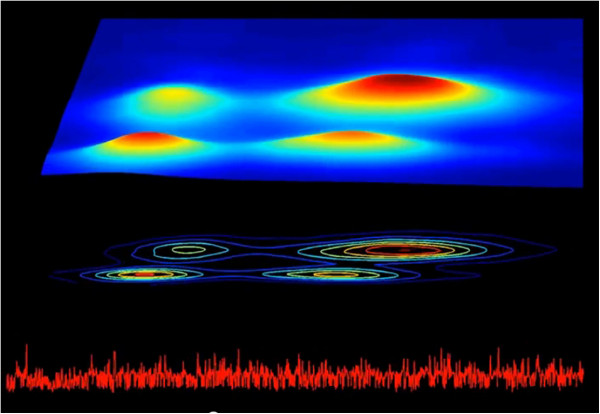
**Snapshot from the D3-Map animation derived from the analysis of a 1200 sec RR interval time series segment of the patient with an atypical pattern of atrial fibrillation.** Top: colourised 3D surface Poincaré map; middle: colourised 2D contour map; bottom: interbeat interval time series.

In contrast, for the second patient with chronic atrial fibrillation (dataset a2nn from http://www.physionet.org/challenge/chaos/), the contour map (Figure 7) shows four loci of high density consistent with a more structured pattern of ventricular response, which are not readily discerned from the time series. The structure centered around (0.4, 0.4) sec in the contour map indicates that the RR intervals fluctuate around 0.4 sec for at least three consecutive beats. Similarly, the structure centered around (0.8, 0.8) sec indicates that the RR intervals fluctuate around 0.8 sec for at least three consecutive beats. The changes from 0.4 to 0.8 and from 0.8 to 0.4, respectively, generate the structures centered around (0.4, 0.8) and (0.8, 0.4) sec. The D3-Map (snapshot shown in Figure 8) animation confirms the dynamical nature of these fluctuation patterns.

This interesting and anomalous pattern of heartbeat timings during AF illustrated in Figures 7 and 8 has been noted by other investigators [12]. However, its electrophysiologic mechanism (possibly involving dual AV nodal pathways or His bundle alternans) and its bedside (diagnostic and prognostic) implications have not been fully addressed. (Clinical details are not available in the two cases presented here). The D3-Map visualization tool may help foster basic and clinical work on different dynamical subsets of AF, which in current practice are all grouped together.

## Conclusions

We present a new animation method for visualising the temporal evolution of complex signal dynamics. For trainees, such simple-to-construct plots (termed “D3-Maps”) may be used to illustrate technical concepts, such as nonstationarity and multistability, which are difficult to grasp, but essential to understanding the dynamics of physiologic control systems. In addition, the animations may reveal unexpected patterns in data structure, which make the D3-Maps useful in exploratory research, facilitating hypothesis generation and development and testing of mathematical/physiologic models.

### Animations

All animations are available at http://reylab.bidmc.harvard.edu/.D3Map/.

Animation 1 (Sleep2D.avi) Movie of the 2D contour Poincare plots. Each frame shows both the colourised 2D contour plot and the 500 sec interbeat interval time series segment used to generate it. Movie frames were generated using "RR.dat" dataset (Additional file 1) and "Sleep2D.m" Matlab script (Additional file 2).

Animation 2 (Sleep3D.avi) D3-Map animation. Each frame shows a 500 sec interbeat interval time series segment (bottom) and the corresponding colourised 3D surface Poincaré (top) and colourised 2D contour (middle) maps. Movie frames were generated using "RR.dat" dataset (Additional file 1) and "Sleep3D.m" Matlab script (Additional file 3). Note: The duration of the Sleep2D.avi and Sleep3D.avi movies is 3 min 20 sec, representing approximately 6 hours of heart rate dynamics obtained during sleep. A 500 sec moving window shifted 10 sec at a time was used to select the data segments displayed in each frame.

Animation 3 (Typical_AF_2D.avi) Movie of the 2D contour Poincare plots for a patient with a typical pattern of atrial fibrillation. Each frame shows both the colourised 2D contour plot and the 1200 sec interbeat interval time series segment used to generate it. Movie frames were generated using "Typical_AF.dat" dataset (Additional file 4) and "Typical_AF_2D.m" Matlab script (Additional file 5).

Animation 4 (Typical_AF_3D.avi) D3-Map animation derived from the analysis of the RR interval time series of a patient with a typical pattern of atrial fibrillation. Each frame shows a 1200 sec interbeat interval time series segment (bottom) and the corresponding colourised 3D surface Poincaré (top) and colourised 2D contour (middle) maps. Movie frames were generated using "Typical_AF.dat" dataset (Additional file 4) and "Typical_AF_3D.m" Matlab script (Additional file 6).

Animation 5 (Atypical_AF_3D.avi) Movie of the 2D contour Poincare plots for a patient with an atypical pattern of atrial fibrillation. Each frame shows both the colourised 2D contour plot and the 1200 sec interbeat interval time series segment used to generate it. Movie frames were generated using "Atypical_AF.dat" dataset (Additional file 7) and "Atypical_AF_2D.m" Matlab script (Additional file 8).

Animation 6 (Atypical_AF_3D.avi) D3-Map animation derived from the analysis of the RR interval time series of a patient with an atypical pattern of atrial fibrillation. Each frame shows a 1200 sec interbeat interval time series segment (bottom) and the corresponding colourised 3D surface Poincaré (top) and colourised 2D contour (middle) maps. Movie frames were generated using "Atypical_AF.dat" dataset (Additional file 7) and "Atypical_AF_3D.m" Matlab script (Additional file 9).

Note: The duration of the Typical_AF_2D.avi and Typical_AF_3D.avi movies is 56 sec, representing approximately 24 h of heart rate dynamics. The duration of the Atypical_AF_3D.avi, and Atypical_AF_3D.avi movies is 47 sec, representing approximately 20 h of heart rate dynamics. A 1200 sec moving window shifted 300 sec at a time was used to select the data segments displayed in each frame of the movies. All Matlab scripts listed above use dscatter2 Matlab function (Additional file 10) to calculate smoothed Poincare map.

## Endnotes

^a^The Poincaré plot is sometimes referred to as a Lorenz plot, a delay map, or a return map.

^b^Matlab code for creating Poincaré plot from the sequence of RR intervals is listed below:

data = load('RR.dat’); % load the Additional file 1 with the RR time series

RR = data(:,2);

RRx = RR(1:end-1);

RRy = RR(2:end);

^c^Due to the normalisation, the height of the 2D histogram is not exactly equivalent to the probability density, but represents a relative occupancy of the phase space mapped onto the [0:1] interval.

^d^Available at http://www.mathworks.com/matlabcentral/fileexchange/8430-flow-cytometry-data-reader-and-visualisation.

^e^A colourmap is defined as a specific chromatic sequence, which is linearly mapped onto the [0:1] interval, so any number between 0 and 1 corresponds to a particular colour.

^f^Since we cannot see the “back” of the 3D surface Poincaré plot, we included its 2D contour Poincaré plot in each figure.

^g^We opted to create animations using stand-alone specialised software, rather than Matlab see Appendix in ref. [13]. Specialised multimedia editing software has additional options for post-processing, such as compression algorithms, annotation editing (e.g., adding extra frames with a movie title, explanatory text or subtitles), as well as audio (e.g., narrations or music).

## Abbreviations

D3-Map: Dynamical density delay map; HR: Heart rate.

## Competing interests

The authors declare that they have no competing interests.

## Authors’ contributions

The conceptualization and initial implementation of the dynamical density delay map method were developed by AB, MDC and ALG. All four authors contributed to the refinement of the method, analysis of the findings and to the composition and editing of the manuscript. All authors have read and approved this manuscript.

## Pre-publication history

The pre-publication history for this paper can be accessed here:

http://www.biomedcentral.com/1472-6947/14/6/prepub

## Supplementary Material

Additional file 1 **(RR.dat).** ASCII file with the cardiac interbeat (RR) intervals obtained from the electrocardiographic recording of a healthy subject during sleep (~6 hour long). This dataset was used to create Sleep2D.avi and Sleep3D.avi movies. Click here for file

Additional file 2 **(Sleep2D.m).** Matlab script used to generate the frames of the Sleep2D.avi movie. Click here for file

Additional file 3 **(Sleep3D.m).** Matlab script used to generate the frames of the Sleep3D.avi movie. Click here for file

Additional file 4 **(Typical_AF.dat).** ASCII file with the cardiac interbeat (RR) intervals obtained from the electrocardiographic recording of a subject with atrial fibrillation. Click here for file

Additional file 5 **(Typical_AF_2D.m).** Matlab script used to generate the frames of the Typical_AF_2D.avi movie. Click here for file

Additional file 6 **(Typical_AF_3D.m).** Matlab script used to generate the frames of the Typical_AF_3D.avi movie. Click here for file

Additional file 7 **(Atypical_AF.dat).** ASCII file with the cardiac interbeat (RR) intervals obtained from the electrocardiographic recording of a subject with atrial fibrillation, whose contour and D3 maps show atypical dynamical patterns. Click here for file

Additional file 8 **(Atypical_AF_2D.m).** Matlab script used to generate the frames of the Atypical_AF_2D.avi movie. Click here for file

Additional file 9 **(Atypical_AF_3D.m).** Matlab script used to generate the frames of the Atypical_AF_3D.avi movie. Click here for file

Additional file 10 **(dscatter2.m).** Matlab function that creates a scatter plot coloured by density. This is a modified version of the original dscatter function http://www.mathworks.com/matlabcentral/fileexchange/8430-flow-cytometry-data-reader-and-visualization/content/dscatter.m. All Matlab scripts listed above use this function. Click here for file
